# TYPES OF APPLICATIONS AND PUTTING YOUR BEST FOOT FORWARD

**DOI:** 10.1093/geroni/igz038.765

**Published:** 2019-11-08

**Authors:** Dana Plude, Dana Plude, Elia Femia

**Affiliations:** 1 National Institute on Aging/National Institutes of Health, Bethesda, Maryland, United States; 2 National Institute on Aging, Bethesda, Maryland, United States; 3 National Institutes of Health, Bethesda, Maryland, United States

## Abstract

The majority of peer review is conducted through the NIH Center for Scientific Review (CSR), which works closely with the institutes and centers who ultimately fund projects of high scientific merit and high potential impact. CSR conducts the review of 90% of R01s, 85% of Fellowships, and 95% of SBIR applications as well as many other research and training opportunity activities. The playing field for successful funding from NIH is highly competitive. Understanding about different application types, who to talk to about your application, finding the right review panel, and learning about the policies pertaining to review are important steps in preparing an application. In this presentation, learn about the important aspects of the grant submission process and how CSR conducts the review of application in close coordination with NIH’s 24 institutes and centers.

